# Residential aged care residents and components of end of life care in an Australian hospital

**DOI:** 10.1186/s12904-018-0337-x

**Published:** 2018-06-09

**Authors:** Laurence Jee Peng Leong, Gregory Brian Crawford

**Affiliations:** 1 Specialist Palliative Care Service - South, Tasmanian Health Service, 1st Floor, Peacock Building, Repatriation Centre, 90 Davey Street, Hobart, TAS 7000 Australia; 2 Northern Adelaide Palliative Service, Modbury Hospital, 41-69 Smart Rd, Modbury, South Australia 5092 Australia; 3 Discipline of Medicine, The University of Adelaide, Adelaide, South Australia 5005 Australia

**Keywords:** Residential facilities, Hospitalization, Terminal care, Advance care planning, Dementia

## Abstract

**Background:**

With ageing of Australians, the numbers of residential aged care (RAC) residents is rising. This places a spotlight on decisions about appropriate care for this population, including hospitalisation and end-of-life (EOL) care. The aim was to study a sample of RAC residents who attended and died in hospital, to quantify measurable components of EOL care so as to describe the extent of palliative care required.

**Methods:**

A retrospective case-note review of hospital records was conducted in Adelaide, Australia. Participants were 109 RAC residents who attended from July 2013 to June 2014 and died in hospital.

Measurements were advance care planning, health care input from the RAC facilities to hospital and components of EOL care. Residents with and without advanced dementia were compared.

**Results:**

Advance care directives (ACDs) were present from 11 to 50%, and advance care plans (ACPs) at 60%. There were more ACPs, resuscitation orders (for/against) and do-not-hospitalise orders in residents with advanced dementia than those without. General practitioner (GP) and extended care paramedic (ECP) input on decisions for hospital transfer were 30% and 1 %. Mean hospital stay to death was 5.2 days. For residents admitted under non-palliative care teams, specialist palliative care (SPC) was needed for phone advice in 5%, consultation in 45%, transfer to palliative care unit in 37%, and takeover by SPC team in 19%. Mean number of documented goals-of-care discussions with family/caregiver was 1.7. In the last 3 days of life, the mean daily number of doses of EOL medications was 4.2. Continuous subcutaneous infusion was commenced in 35%.

**Conclusion:**

Staff in RAC need to be adequately resourced to make complex decisions about whether to transfer to hospital. RAC nurses are mainly making these decisions as GP and ECP input were suboptimal. Ways to support nurses and optimise decision-making are needed. Advance care planning can be improved, especially documentation of EOL wishes and hospitalisation orders.

By describing the components of EOL care, it is hoped providers and policy makers have more information to assist with making decisions about what is the most appropriate care for this population.

## Background

In Australia, a residential aged care facility (RACF) is a *special-purpose facility which provides accommodation and support, including assistance with day-to-day living, intensive forms of care, and assistance towards independent living, to frail and aged residents (predominantly 65 years or over)* [1]. Internationally, RACFs are variously known as nursing homes, long-term care facilities or other names depending on the country. The term RACF is used interchangeably in this article. With population ageing in Australia, the numbers of residential aged care (RAC) residents is rising. In 2016, about 3.7 million Australians were aged 65 years or over [2]. In 2011, about 4% of Australians aged 65 years or over resided in RACFs [3].

Studies of hospitalisation of RAC residents internationally have shown that rates vary from 9 to 60% annually [4]. Predictors of hospitalisation have been studied [5, 6] and reviewed [7]. Factors increasing RAC residents’ likelihood of hospitalisation were socio-demographics (older, male, shorter length of stay), health characteristics (higher burden of chronic disease, polypharmacy, functional disability and various acute problems, particularly infections), certain care practices (physical restraints, pressure sores, feeding tubes, urinary catheters), RACF provider attitudes (overburdening of staff) and financial reimbursement issues. Factors decreasing hospitalisation were presence of dementia and neurological disorders [6, 7], advance care directives (ACDs) and use of hospice, a specific model in the United States of America (USA). Availability of certain medical interventions (intravenous therapy, respiratory therapy) and support services (on-site radiology, laboratory testing) had conflicting associations with hospitalisation. Greater staff resource, in terms of nurses and physicians, showed conflicting data in the review [7] but was associated with decreased hospitalisation in other studies [5, 6].

For RAC residents, hospital transfer decisions are complex. Studies have attempted to measure whether hospitalisations were inappropriate or avoidable [8–11]. Estimated rates of avoidable hospitalisation vary from 19 to 67% [4]. Reviews of measures of appropriateness of hospital transfer have described studies using various tools and their underlying frameworks [12, 13]. These studies had methodological shortcomings, including being based on expert opinion and retrospective in nature (not tested at point-of-care), and the tools lacked comprehensiveness and generalisability.

To the best of our knowledge, there is no literature that directly focuses on the benefits of hospitalisation for RAC residents. However, there is recent literature on the benefits of hospitalisation for those with palliative care need [14, 15]. Conversely, the documented harms of hospitalisation include delirium, falls, worsening function, pressure sores, hospital-acquired infections, medication misadventure and care inconsistent with patients’ wishes, not to mention the cost to the health system [4, 12, 16, 17]. Observational studies show better outcomes treating infections in the RACF compared to hospital [18, 19]. For residents with advanced dementia, compared to a strategy of staying in the RACF, hospitalisation was not cost-effective, especially in the setting of pneumonia [20], and treatment of pneumonia with antibiotics was associated with worse comfort [21].

A systematic review of interventions to reduce hospitalisation of RAC residents [4] showed positive results for advance care planning [22, 23], pneumonia assessment/treatment pathway, mobile geriatric outreach, Hospital in the Nursing Home (HITNH) in combination with advance care planning [23], influenza immunisation and hospice referral (specific to USA). In the Australian advance care planning study, the intervention group showed a statistically significant reduction in mortality [23]. An Irish study showed that advance care planning reduced hospitalisation and overall cost [24]. Three Australian studies have used emergency department (ED) nurses or HITNH to support RACFs [25–27]. Hospital in the Nursing Home has evolved from Hospital in the Home, which are nurse-led programs designed to avoid hospitalisation. These studies have shown reduced ED visits and/or hospital admissions. An advance care planning study in USA, using a video decision-aid in patients with advanced dementia, showed improved quality of communication and reduced hospitalisation [28]. The Aged Residential Care Healthcare Utilisation Study (ARCHUS), a nurse-led multidisciplinary geriatric intervention study in New Zealand, showed no effect on hospitalisations [29]. The Regular Early Assessment Post-Discharge (REAP) study, a predominantly physician-led multidisciplinary geriatric intervention study, showed reduced hospital readmissions, ED visits and overall cost [30].

The literature on care provision of RAC residents, in the last months of life and at the time of death, does provide data on hospital use (frequency and duration of hospitalisations, and rate of death in hospital) and associated clinical problems (underlying and necessitating hospital admissions) [31–37]. In a South Australian (SA) study of a general sample of RAC residents admitted to a metropolitan hospital over 6 months, 4% died during the admission [31]. In a German study in the last 12 months of life, about 58% of RAC residents were admitted to hospital at least once [32], compared to about 40% in a Canadian study in the last 6 months of life [33], and about 34% in an English study in the last 1 month of life [34]. The frequency of death in hospital of RAC residents is reported to be about 29% in Germany [32], 27% in England [34], 19% in Canada [33] and 9% in New Zealand [35]. In Australia during 2010–11, about 18% of RAC residents died outside of the RACF, i.e. the majority in hospital [36]. Measurement of care provision by specific health care providers has been undertaken in the English study [34] and a Norwegian study, which looked at RAC residents who died within 48 h of hospitalisation [37]. This Norwegian descriptive study was the first to measure care provision by providing a detailed account of specific care interventions (diagnostic tests, life prolonging and palliative interventions). Our study adds to this by providing a more detailed description of the specific palliative end-of-life (EOL) interventions.

Advance care planning in RACFs has been studied in SA [38]. Advance care directives are government legislated documents completed by patients with decision capacity. The ACDs during this study are in Table 1. Advance care plan (ACP) documents are not legislated. Whilst every effort should be made to involve the patient in decision-making, ACPs can be used even when patients have lost decision capacity and can be completed by health professionals in discussion with family/caregivers through shared decision-making. In the past, use of advance care planning documents in SA was low [38]. It was interesting to compare usage with this study sample.

**Table 1:** Advance care directives in South Australia before 2014 and their function

Advance care directive document	Decision focus
Anticipatory Direction	Wishes for End-of-Life Care
Medical Power of Attorney	Proxy for Medical Decisions
Enduring Power of Guardian	Proxy for Lifestyle and Medical Decisions
Enduring Power of Attorney	Proxy for Financial and Property Decisions

The main aim of this study was to describe a sample of RAC residents who died in hospital to quantify easily measurable components of end-of-life-care (EOLC). By describing the extent of palliative care required, it may provide some information that assists with RAC resident transfer decisions between the RACF and hospital. For this study, EOLC refers to care in the last few days to weeks of life. To test the hypothesis that the advance care planning and components of EOLC may be different between residents with and without advanced dementia, comparisons were conducted.

## Methods

### Design

This study was a retrospective case-note review of hospital records and was predominantly descriptive. Cases were from 27 RACFs surrounding a hospital in the suburbs of Adelaide, SA. This facility is an acute/subacute care hospital that provides inpatient, outpatient and emergency services to about 200,000 people. Inclusion criteria were RAC residents who attended this hospital (ED and hospital admissions) from July 2013 to June 2014, and died in hospital. Outpatient attendances were excluded.

### Outcome measures

A data sheet was designed that detailed:

1. Demographics, comorbidities and illness trajectories. The types of care were categorised as high/low, permanent or respite (up to 63 days/year), or transitional care package (slow stream rehabilitation with goal of returning home). The Global Deterioration Scale is a seven point rating scale to measure the degree of impairment of persons with Alzheimer’s dementia. A rating of seven defines advanced dementia and is characterised by poor verbal ability, inability to mobilise and requiring assistance with feeding and toileting [39]. Illness trajectories were based on Lunney et al. [40] and used similarly to Menec et al. [32]. “Terminal illness” included cancer, motor neurone disease and end-stage renal disease; “organ failure” included heart failure, stroke, chronic obstructive pulmonary disease and ischaemic heart disease; “frailty” included dementia and multiple sclerosis. An “other” group was included.

2. Advance care directives and ACPs prior to hospital attendance, including resuscitation and hospitalisation orders.

3. Logistics of presentations and hospital stay, including whether the time of presentations were during working hours (Monday to Friday, 9 am to 5 pm) or after-hours.

4. Input of general practitioner (GP) or family physician**,** ambulance, ED and hospital teams. At this hospital, the specialist palliative care (SPC) team consisted of both an inpatient multi-disciplinary palliative care unit (PCU) and a consultation liaison team consisting of nurse and physician.

5. Components of EOLC were SPC input, number of goals-of-care (GOC) discussions with family/caregivers and EOL medication use. Discussions with family/caregivers were in person or by phone, and there was documentation in the hospital record of a conversation about the type of medical care that was appropriate. End-of-life medication use was measured per day during the last 3 days of life. These medications were for symptom control and were recorded in the regular, prn (if required) or stat (immediate) sections of the medication chart. They could be administered via any route.

### Data sources

Patient list and administrative data were obtained from the hospital database. The remainder of the data was obtained from hospital records.

### Study size

One hundred and nine patients were identified from July 2013 to June 2014 and all case notes reviewed.

### Statistics

The data was summarised using standard descriptive statistics. To compare residents with/without advanced dementia, a Wilcoxon test was used to compare differences of means and a Pearson’s Chi-square test to compare differences of proportions. Statistical analysis was completed using SAS v9.4 (SAS Institute Inc., Cary, NC, USA)**.**

## Results

The total number of hospital attendances (excluding outpatient visits) over 12 months was 42,142. The number of RAC resident attendances was 1545 (3.7%). The number of RAC residents who died in hospital was 109 (7.1% of RAC resident attendances). The number of RAC residents who died within 1 week of discharge to the RACF was 66 (4.3% of RAC resident attendances).

The study sample demographics were typical of an Australian RAC population [41] (Table 2). There were 109 hospital attendances by RAC residents from 27 RACFs, of which 99 (91%) were from RACF to ED. Six (6%) residents were admitted directly to the ward, three (3%) were transferred from a neighbouring acute tertiary hospital in Adelaide, and one (1%) was admitted from an outpatient clinic appointment. Discounting inter-hospital transfers, there were 106 attendances from RACF to hospital. Of these, 71 (67%) were after-hours.

**Table 2:** Demographics of residential aged care residents prior to hospital attendance

		Number (%)	Mean (SD)
Sex	Men	43 (39)	
	Women	66 (61)	
Age			84.3 (8.3)
Age Group	< 60	2 (2)	
	60–69	3 (3)	
	70–79	18 (17)	
	80–89	57 (52)	
	90–99	29 (27)	
Principal Medical Problem(s)	Non-Advanced Dementia	34 (31)	
	Advanced Dementia	25 (23)	
	Cardiac	52 (48)	
	Respiratory	29 (27)	
	Neurological	31 (28)	
	Cancer	13 (12)	
	Other	9 (8)	
Trajectory	Organ Failure	70 (64)	
	Frailty	61 (56)	
	Terminal	13 (12)	
	Other	6 (6)	
Types of Care	High Level	97 (89)	
	Low Level	7 (6)	
	Permanent	101 (93)	
	Respite	3 (3)	
	TCP	5 (5)	
Specialist Palliative Care	Known to Community Palliative Care	12 (11)	
Total		109	

*SD* Standard Deviation, *TCP* Transitional Care Package

Table 3 summarises the advance care planning. For ACDs in the sample of 109, 72 (66%) had some documentation. Documenting EOL wishes (Anticipatory Direction) was present in 12 (11%). For residents with/without advanced dementia, there was no difference in documentation of ACDs. For ACPs, 65 (60%) had some documentation. There was more documentation of ACPs for residents with than without advanced dementia (*p* = 0.005).

**Table 3:** Various advance care planning documents of residential aged care residents prior to hospital attendance

Measures		Total	Advanced Dementia (AD)	Without Advanced Dementia (WAD)	AD vs WAD
		Number (%)	Number (%)	Number (%)	*p* value
Advance Care Directives	Documentation	72 (66)	16 (64)	56 (67)	*p* = 0.805
	EPOA	54 (50)	13 (52)	41 (49)	*p* = 0.779
	EPOG	40 (37)	8 (32)	32 (38)	*p* = 0.579
	MPOA	10 (9)	2 (8)	8 (10)	*p* = 0.817
	EPOG or MPOA	49 (45)	10 (40)	39 (46)	*p* = 0.571
	Anticipatory Direction	12 (11)	1 (4)	11 (13)	
Advance Care Plans	Documentation	65 (60)	21 (84)	44 (52)	*p* = 0.005
	RACF Specific	30 (28)	9 (36)	21 (25)	*p* = 0.280
	GPCP	33 (30)	12 (48)	21 (25)	*p* = 0.028
	SOC	2 (2)	0 (0)	2 (2)	
	Other	1 (1)	1 (4)	0 (0)	
Resuscitation Orders	Documentation	70 (64)	21 (84)	49 (58)	*p* = 0.019
	Not for Resus	67 (61)	21 (84)	46 (55)	*p* = 0.008
	For Resus	3 (3)	0 (0)	3 (4)	
Hospitalisation Orders	Documentation	27 (25)	8 (32)	20 (24)	*p* = 0.411
	Do Not Hospitalise	15 (14)	7 (28)	8 (10)	*p* = 0.019
	Do Hospitalise	12 (11)	1 (4)	11 (13)	
Total		109	25	84	

*EPOA* Enduring Power of Attorney, *EPOG* Enduring Power of Guardian, *MPOA* Medical Power of Attorney, *RACF* Residential Aged Care Facility, *GPCP* Good Palliative Care Plan (from Palliative Care SA), *SOC* Statement of Choices (from Respecting Patient Choices Program)

For overall resuscitation orders (for/against), 70 (64%) had documented preferences. Comparing residents with/without advanced dementia, those with advanced dementia had more overall resuscitation orders (*p* = 0.019) and more not-for-resuscitation (NFR) orders (*p* = 0.008). For overall hospitalisation orders (for/against), 27 (25%) had documented preferences. Residents with advanced dementia had no difference in overall hospitalisation orders but had more do-not-hospitalise (DNH) orders (p = 0.019) compared with residents without advanced dementia.

Whilst at the RACF, 100 residents had potential for GP input in the decision about hospital transfer. In the hospital record, there was documentation of GP input in 30 (30%) transfers. Of these, 22 (73%) had input from the usual GP and seven (23%) from a locum GP. General practitioner assessments were face-to-face in 19 (63%), telephone in six (20%) and not documented in five (17%).

All residents were transferred from RACF to hospital by ambulance. In metropolitan Adelaide, extended care paramedics (ECPs; ambulance officers with expanded scope of practice) are available to provide assistance with hospital avoidance when appropriate. Only one resident had ECP input.

In Australia, the Australasian Triage Scale rates priority and waiting time from one to five [42]. For the 99 residents who attended ED, ten (10%) were category one, 28 (28%) were category two, 60 (61%) were category three and one (1%) was category four. For category three, patients should be seen within 30 min. In this study, the mean time to physician assessment was 10.3 min (range 0 to 44 min). The mean length of stay (LOS) in ED was 7.7 h (range 1.1 to 47.7 h).

Of the 109 RAC residents who attended hospital, eight (7%) either died in ED or were dead-on-arrival. The 101 residents admitted into hospital are detailed in Table 4. Of these, 14 (14%) residents were admitted directly under the SPC team and 87 (86%) under non-palliative care teams, the majority under general medicine. The mean hospital LOS (admission to death) was 5.2 days. There was no difference between residents with/without advanced dementia.

**Table 4:** Hospital admission characteristics of residential aged care residents, including residents with and without advanced dementia

Measures		Total		Advanced Dementia		Without Advanced Dementia	
		Number (+/− %)	Mean (SD, Range)	Number (+/− %)	Mean (SD, Range)	Number (+/− %)	Mean (SD, Range)
For Patients Admitted		101		22		79	
Admission Team	General Medicine	76 (75)		21 (95)		55 (70)	
	Surgery	3 (3)		0 (0)		3 (4)	
	Orthopaedics	3 (3)		0 (0)		3 (4)	
	Palliative Care	14 (14)		1 (5)		13 (16)	
	ICU/HDU	5 (5)		0 (0)		5 (6)	
Admission	Length of Stay (Days)		5.2 (5.3, 0.1–33.1)		3.5 (2.5, 0.4–11.8)		5.7 (5.7, 0.1–33.1)
	Advanced Dementia vs Without Advanced Dementia					*p* = 0.330	
For Patients with Admission Completed		103		24		79	
Resuscitation Order	Documented	92 (89)		23 (96)		69 (87)	
	Not Documented	11 (11)		1 (4)		10 (13)	
If Resuscitation Documented	Not for Resuscitation	89 (97)		23 (100)		66 (96)	
	For Resuscitation	3 (3)		0 (0)		3 (4)	
For Patients Admitted		101		22		79	
Number of Treating Teams	One	77 (76)		18 (82)		59 (75)	
	Two	22 (22)		4 (18)		18 (23)	
	Three	2 (2)		0 (0)		2 (2)	
Treating Teams	General Medicine	82 (81)		21 (95)		61 (77)	
	Surgery	3 (3)		0 (0)		3 (4)	
	Orthopaedics	3 (3)		0 (0)		3 (4)	
	Palliative Care	31 (31)		5 (23)		26 (33)	
	ICU/HDU	9 (9)		0 (0)		9 (11)	

*SD* Standard Deviation, *ICU/HDU* Intensive Care Unit/High Dependency Unit

Measures of components of EOLC are in Table 5. Input from SPC was evaluated in 87 RAC residents admitted under non-palliative care teams. Phone advice was sought from SPC in four (5%) cases and referral for SPC consultation was requested in 38 (44%). Transfer to the PCU occurred in 32 (37%) cases and transfer of care to the SPC team occurred in 18 (21%). There was no difference in SPC input for residents with/without advanced dementia.

**Table 5:** Measures of components of end of life care of hospitalised residential aged care residents

Measures	Total		Advanced Dementia (AD)		Without Advanced Dementia (WAD)		AD vs WAD
	Number (+/− %)	Mean (SD, Range)	Number (+/− %)	Mean (SD, Range)	Number (+/− %)	Mean (SD, Range)	*p* value
For Admitted under SPC	14		1		13		
PCU Length of Stay		6.7 (8.4, 1.3–33.1)		2.2 (N/A)		7.0 (8.6, 1.3–33.1)	
For Admitted under Non SPC	87		21		66		
SPC Advice Only	4 (5)		0 (0)		4 (6)		*p* = 0.568
SPC Consult Referral	38 (44)		10 (48)		28 (42)		*p* = 0.676
Patient Seen by SPC	33 (38)		10 (48)		23 (35)		
Patient Died Before Seen	5 (6)		0 (0)		5 (8)		
Any SPC Involvement	42 (49)		10 (48)		32 (48)		*p* = 0.945
For SPC Consult							
Days to be Seen		0.6 (1.1, 0–3)		0.6 (1, 0–3)		0.6 (1.1, 0–3)	
SPC Take Over	18 (21)		4 (19)		13 (20)		*p* = 1.00
For PCU							
Transfer to PCU	32 (37)		8 (38)		24 (36)		*p* = 0.886
PCU Length of Stay		2.4 (2.6, 0–11.4)		3.0 (1.4, 1.6–5.3)		2.3 (2.9, 0–11.4)	
For All Patients (including Not Admitted)							
Number of Family/Caregiver Goals of Care Discussions		1.7 (1.0, 0–5)		1.5 (0.5, 1–2)		1.7 (1.1, 0–5)	*p* = 0.581
End of Life Medications in Last Three Days							
Opioid Charted	95 (87)		20 (80)		75 (89)		
Opioid Dose(s) per Day		2.5 (1.9, 0–8.7)		2.4 (1.5, 0–5)		2.6 (1.9, 0–8.7)	
Antipsychotic Charted	47 (43)		9 (36)		38 (45)		
Antipsychotic Dose(s) per Day		0.4 (0.7, 0–3)		0.1 (0.2, 0–0.7)		0.4 (0.7, 0–3)	
Pure Antiemetic Charted	51 (47)		8 (32)		43 (51)		
Pure Antiemetic Dose(s) per Day		0.3 (0.6, 0–3)		0.5 (1.1, 0–3)		0.3 (0.5, 0–2)	
Benzodiazepine Charted	80 (73)		16 (70)		63 (75)		
Benzodiazepine Dose(s) per Day		1.4 (1.5, 0–6.3)		1.6 (1.3, 0–4)		1.4 (1.6, 0–6.3)	
Antisecretory Charted	77 (71)		17 (68)		58 (69)		
Antisecretory Dose(s) per Day		1.0 (1.2, 0–5)		1.1 (1.4, 0–4.3)		0.9 (1.2, 0–5)	
Total Doses per Day		4.2 (3.5, 0–18)		4.0 (3.3, 0–11.3)		4.2 (3.6, 0–18)	*p* = 0.867
CSCI Commenced	38 (35)		8 (32)		30 (36)		*p* = 0.732

*SD* Standard Deviation, *SPC* Specialist Palliative Care, *PCU* Palliative Care Unit, *CSCI* Continuous Subcutaneous Infusion

The mean number of documented GOC discussions with family/caregivers was 1.7. There was no difference between residents with/without advanced dementia.

For EOL medication use in the last 3 days of life, charting of opioids was the most common, followed by benzodiazepines, anti-secretory agents, anti-emetics and anti-psychotics. The mean daily number of doses of medications was highest for opioids, followed by benzodiazepines, anti-secretory agents, anti-psychotics and anti-emetics. The mean daily number of doses of all EOL medications was 4.3. A continuous subcutaneous infusion (CSCI) was commenced in 38 (35%) cases. There was no difference in the overall administration of EOL medications between residents with/without advanced dementia.

## Discussion

In terms of advance care planning, participants in this sample appointed legal proxies for financial decisions in 50% and medical decisions in 45%. Documenting wishes for EOLC (Anticipatory Direction) was present in 11%. In the advance care planning study of hospitalised elderly (80 years or over) by Detering et al. [43], of the 154 intervention participants, 70 (45%) completed a written “ACP” (equivalent to ACD). This suggests that documenting wishes for EOLC can be improved. Advance care plans were present in 60% and resuscitation orders in 64% of the sample. Hospitalisation orders (an order detailing a preference about hospitalisation) were present in 25%. It is possible to improve rates of DNH orders as shown by Lamberg et al. [44]. Their retrospective cohort study of patients with advanced dementia in USA found at time of death, rate of DNH orders was 83.8%. This suggests that discussions and recording of hospitalisation orders can be improved. Although this study shows higher rates of ACDs and ACPs compared with an earlier study [38], improving the quality and frequency of advance care planning remains important because it is known to improve the rates of EOL wishes being respected [43], (i.e. improved decision-making), and reduce hospitalisation [22–24, 28].

In terms of participant pre-hospital resource use, GP input occurred in 30% of cases and ECP input in 1%. This data, showing that RAC nurses are responsible for most decisions about hospital transfer due to suboptimal access to physician input, is consistent with the literature [13, 45]. Interventions to improve RAC nurses’ knowledge and skills is associated with reduced hospitalisations [46]. There is interest in developing alternative models to support nurses in decision-making. Using ED nurses and HITNH have been shown to reduce hospitalisations [25–27] but they were only available during working hours in these studies. Extended care paramedics were introduced in metropolitan Adelaide in 2008 and may be of use, especially after-hours. Observational research is being conducted to measure ECP use and hospital avoidance [47] but there are limited intervention trials on the effectiveness of ECPs [48]. In this study, there were residents who could have received ECP care and whether RAC staff are using this option is not known.

In terms of participant in-hospital resource use, for residents admitted under non-palliative care teams, referral for SPC consultation occurred in 44% and phone advice was provided in 5%. In comparison, Le et al. evaluated EOLC and measured SPC consultations for all patients in a large hospital in Melbourne, Australia [49]. They found that, of the patients where death was anticipated, 42% were referred for SPC consultation, which is comparable with this study.

In terms of EOL medications, our study showed in the last 3 days of life, charting of opioids was 87%, benzodiazepines 73% and antipsychotics 43%, which compares favourably with a retrospective cohort study of three RACFs in Norway, that showed on the last day of life, Morphine was charted in 71.4%, Midazolam 55% and Haloperidol 46.9% [50]. In terms of administration of EOL medications, there have been two recent retrospective cohort studies from the Netherlands, one conducted in a PCU [51] and another conducted in hospital, hospice and home settings [52]. These studies of the last days of life, have measured the proportion of use of specific medications, types of medications (palliative vs preventative) and medication classes, the total amount given in milligrams each day and route of administration. Our study adds to this by measuring the actual number of doses of medications each day given in each class, in order to indicate the extent of palliative medication administration in the EOL.

To summarise the components of EOLC for the average resident in this sample, hospital LOS (admission to death) was about 5 days. Less than two GOC discussions with family/caregivers were documented. A little over four doses of medications were required per day in the last 3 days of life. A CSCI was needed in about one third. About half of the residents who were under non-palliative care teams needed SPC input. It is hoped that having specific information about the extent of palliative care required will assist with decision-making, including appropriateness of transfers between RACF and hospital, and feasibility of care in the RAC setting.

Although this study is thought to be the most detailed description of EOL palliative interventions of RAC residents in hospital, it has significant limitations due to study design (retrospective and case-note review). It focuses on care that has been delivered instead of measures such as care need, quality of care or quality of dying. The components of care measured are medico-centric and fail to capture the breadth and complexity of EOLC [53]. Data collection was from hospital records. Measurement of advance care planning was dependent on delivery of the RACF record to hospital. There may be underestimation of presence of advance care planning documents and GP input. The number of GOC discussions with family/caregivers may be underestimated due to inadequate documentation. The findings may be specific to RACFs in an Australian setting and not be generalisable to other locations.

## Conclusion

Decision-making in the care of RAC residents is often complex and challenging. Resourcing RACFs adequately is important, especially for making decisions whether to transfer to hospital. This study reinforces that RAC nurses are mainly making these decisions as GP and ECP input were suboptimal. Ways to optimise decision-making may include quality improvement of RAC nurses and policy measures to maximise input from the usual GP. Alternative models to support RAC staff include greater collaboration with emergency staff and HITNH. Advance care planning plays an important role in decision-making and this study suggests there may be room for improvement, especially documentation of EOL wishes and hospitalisation orders.

This study has described the components of EOLC for hospitalised RAC residents. By describing the extent of palliative care required, it is hoped that providers and policy makers in primary care, RAC, ambulance and hospital services have more information to assist with making decisions about what is the most appropriate care for this population.

## Abbreviations

ACD: Advance care directive
ACP: Advance care plan
CSCI: Continuous subcutaneous infusion
DNH: Do not hospitalise
ECP: Extended care paramedic
ED: Emergency department
EOL: End-of-life
EOLC: End-of-life-care
GOC: Goals-of-care
GP: General practitioner
HITNH: Hospital in the nursing home
LOS: Length of stay
NFR: Not for resuscitation
PCU: Palliative care unit
RAC: Residential aged care
RACF: Residential aged care facility
SA: South Australia
SPC: Specialist palliative care
